# Editorial: Beyond Antimicrobials: Non-traditional Approaches to Combating Multidrug-Resistant Bacteria

**DOI:** 10.3389/fcimb.2019.00343

**Published:** 2019-10-11

**Authors:** Natalia V. Kirienko, Laurence Rahme, You-Hee Cho

**Affiliations:** ^1^Department of BioSciences, Rice University, Houston, TX, United States; ^2^Department of Surgery, Massachusetts General Hospital, Harvard Medical School, Boston, MA, United States; ^3^Department of Microbiology, Harvard Medical School, Boston, MA, United States; ^4^Shriners Hospitals for Children Boston, Boston, MA, United States; ^5^Department of Pharmacy, College of Pharmacy and Institute of Pharmaceutical Sciences, CHA University, Seongnam, South Korea

**Keywords:** antivirulence, phage (bacteriophage), antimicrobial peptide (AMP), quorum sensing (QS), antimicrobial resistance (AMR), novel therapeutic agents, drug repurposing and repositioning

Since the time of Galen and Hippocrates, medicine has had a slow progression, with each new advance improving the length and quality of patients' lives. By the nineteenth century, medical science had even begun to understand the concept of vaccinations and had started to make substantial inroads into the prevention of communicable diseases. Despite this, diagnosis with a bacterial infection frequently remained tantamount to a death sentence. With treatment essentially limited to supportive care, patients were left to either recover or die. But in the twentieth century, the discovery of antimicrobials changed this. It is not an overstatement to say that medical science changed forever, because this allowed the development of countless medical advances that fall under the classification of internal medicine. Ranging from simple surgeries like the removal of an inflamed appendix to the complete replacement of whole organs and organ systems, not to mention the placement of various medical devices that have improved the lives of countless patients.

Despite this, infectious diseases, including those caused by bacteria, remain the leading cause of premature death worldwide. Problematically, this trend looks as though it will worsen, as both the number and the proportion of clinically relevant bacterial strains and species exhibiting antimicrobial resistance is on the rise. For the ESKAPE pathogens (i.e., *Enterococcus faecium, Staphylcoccus aureus, Klebsiella pneumonia, Acinetobacter baumanii, Pseudomonas aeruginosa*, and *Enterobacter* spp.), not only are single and multidrug resistance common, but pandrug resistance has begun to be clinically observed (Mulani et al., 2019). Overall, for the first time in nearly a century, the spread of antimicrobial resistance has begun to lead to regressions in treatment options and the re-emergence of formerly treatable infections as real threats to community health. While the public remains largely complacent to the threat, experts in healthcare warn of the “galloping hoofbeats of the horsemen of the apocalypse” and the very real possibility that voluntary medical procedures will become a historical phenomenon within the next century, even in the developed world (Projan, 2003).

Further complicating matters, antimicrobial drug discovery has dramatically slowed over the last 20 years, with a paucity of new treatments on the market or in development pipelines. The most obvious problem is scientific. Using the same methods to screen the same libraries leads to the same treatments, which are now ineffective. Finding new methods and new libraries is one option, but it is laborious and requires a leap of faith that the new method will be effective. A more serious problem is economic (Ventola, 2015; Renwick and Mossialos, 2018). For large pharmaceutical companies, the return on investment for antimicrobials tends to be much lower than for lifestyle medicines, which are intended for disease maintenance rather than a cure. For antimicrobials, maintenance is often impractical or impossible. Moreover, identification, development, design, regulatory approval, and marketing are necessary steps for a large pharmaceutical corporation to get a new treatment into clinics for patients. These steps can take from 5 to 20 years. Meanwhile, antimicrobial resistance frequently arises in less than five, particularly when antimicrobial stewardship (i.e., limiting antimicrobial use to last-ditch cases, preventing agricultural or household cleaner use, etc.) is not stringently maintained (Rice, 2018). Worse yet, pricing for antibiotics tends to be lower than for other drugs that have such outsized impacts on morbidity and mortality (e.g., cancer drugs). Finally, the same stewardship programs limit antibacterial use to prolong clinical utility, also artificially curtailing the market size. Cumulatively, these effects result in a monetary loss for the company.

A final obstacle is the incredible regulatory hurdles, particularly in the US, that stand in the way of companies and organizations willing to take on the burden (Metlay et al., 2006). For example, due to the transience of bacterial infections, it is often difficult to find enough patients for large-scale human trials. Another difficulty is the approval process (particularly the US FDA, the EMA has been generally more tractable) that places an undue burden of proof on pharmaceutical companies (e.g., patients only qualify as having been successfully treated if the causative bacterium is identified in complex infections like bacterial pneumonia, difficulty in demonstrating superiority of new treatments to existing therapy instead of non-inferiority to current treatments, etc.). Regulatory and legislative efforts have been initiated in the past 15 years to combat these trends, but rectification has occurred at a glacial pace (Humphries et al., 2018; Sfeir, 2018).

Clearly, it behooves us to seek out alternative mechanisms of treatment to address this growing gap. Therefore, currently there is an increased interest in alternative approaches to the treatment of drug-resistant bacteria. A number of these strategies are presented in this Research Topic.

## Anti-Virulence Approaches

To mitigate the resistance emergence, one increasingly viable option is the discovery and development of the therapeutic chemicals that target bacterial virulence, rather than bacterial growth. In most cases (with the notable exception of immune-related pathology activated by structural components), the mere presence of bacterial cells is insufficient to trigger disease. Instead, pathogenic microorganisms produce various virulence factors that are responsible for the damage inflicted on the host. There is mounting evidence that these pathogenic determinants are viable pharmaceutical targets; treatments that compromising one or more of these factors (i.e., anti-virulence or antipathogenic) can often largely, or even completely, mitigate disease. Anti-virulence drugs should reduce antibiotic use and, ultimately, decrease the development of antibiotic resistance, as they should not impose strong selective pressure on bacteria that favors the evolution of mechanisms of resistance and persistence. Additionally, because they do not affect bacterial cell viability, they should not disrupt beneficial microbiota. Anti-virulence compounds could serve as alternatives or adjuncts to traditional antibiotics and to potentiate their efficacy, generating even more effective treatment options in particular against multi-drug resistant pathogens and potentially viable options against pan-drug resistant bacteria.

A number of articles utilizing this approach are reported in this Research Topic. One promising virulence-associated target is the quorum-sensing (QS) systems utilized by several clinically relevant pathogens (Rutherford and Bassler, 2012; Schuster et al., 2013; Castillo-Juarez et al., 2015). Singh et al. report on the inhibitory activity of 3-benzyl-hexahydro-pyrrolo[1,2-a]pyrazine-1,4-dione on QS in *Pseudomonas aeruginosa*. Interestingly, treatment also altered biofilm architecture, compromising bacterial adherence and biofilm development. Fong et al., report on itaconimide, another novel inhibitor of Pseudomonal QS and biofilm. Perhaps unsurprisingly, inhibition of biofilm formation is another common target for the development of anti-virulent compounds (Maura et al., 2016; Francois et al., 2017; Defoirdt, 2018; Salam and Quave, 2018). This approach can also be used against opportunistic fungi. For example, Lee et al. describe antibiofilm activity for 6-gingerol and 6-shogaol against *Candida albicans*. These two molecules prevented cell aggregation and inhibited the expression of several biofilm-related genes.

Both QS signaling and biofilm contribute to chronic infections. They are associated with complex regulatory switches that control large numbers of genes whose activity alters the lifestyle of the pathogen to support long-term microbial presence within a host. This is in contrast to more stereotypically acute virulence determinants (e.g., the Type 3 Secretion System, proteases, lipases, toxins, etc.) that cause consequences for which microbial presence is less relevant. Dong et al. found that the flavonoid morin inhibits the hemolytic activity of aerolysin, protecting catfish from infection with *Aeromonas hydrophila*.

Generally, drug discovery attempts have targeted chronic virulence factors. A notable exception to this is the Pseudomonal siderophore pyoverdine. Unlike many acute virulence determinants, pyoverdine plays a diverse and multifactorial role in pathogenesis, including iron acquisition (necessary for production of other virulence factors, including biofilm, and for bacterial growth) and is a transcriptional regulator of several other virulence factors and toxins (Ochsner et al., 1996; Wilderman et al., 2001; Lamont et al., 2002). In addition, it is directly cytotoxic (Kang et al., 2018). Previously, preventing pyoverdine biosynthesis has been shown to attenuate virulence in a variety of hosts, including invertebrates and mice (Meyer et al., 1996; Takase et al., 2000; Minandri et al., 2016). Here, Imperi et al. examined the limitations of this approach by selecting for *P. aeruginosa* mutants resistant to one class of these inhibitors, fluoropyrimidines (Imperi et al., 2013; Kirienko et al., 2016). To circumvent this form of resistance, Kirienko et al. identified small molecules that directly inhibit pyoverdine function, rather than its production. Interestingly, these molecules are also effective against multidrug-resistant isolates of *P. aeruginosa* collected from patients with cystic fibrosis (Kang et al., 2019).

Frequently the strongest effects for anti-virulence molecule are in combination with other treatments. For example, Tharmalingam et al. demonstrated that 4-(1,3-dimethyl-2,3-dihydro-1H-*S*-benzimidazol-2-yl)phenol (BIP) inhibited several virulence factors of methicillin-resistant *Staphylococcus aureus* (MRSA). BIP treatment decreased MRSA virulence and sensitized bacteria to macrophage-dependent killing. Marini et al. showed that *Cannabis sativa* extracts reduced motility and biofilm formation in the food-borne pathogen *Listeria monocytogenes*. Martínez et al. reviewed a number of promising anti-virulence strategies, including disassembly of functional microdomains in bacterial membranes and toxin neutralization.

## Alternative Approaches: Drug Repurposing, Combination Therapies, and Non-Conventional Bactericides

Regulatory hurdles are another stumbling block in drug discovery. Researchers have begun to propose and implement innovative strategies to practically reduce time and costs for the drug development processes. Repositioning or repurposing already approved drugs is an approach that has recently gained momentum, with the hypothesis being that the compounds may have new modes of action for which the resistance has yet to develop. Similarly, combining two or more compounds with different or synergistic mechanisms is another alternative approach to improve the efficacy of the currently available antibiotic regimens and may increase the success rate of drug repositioning as well.

In this collection, Miró-Canturri et al. reviewed the current state of knowledge regarding drug repurposing strategies for the treatment of bacterial and fungal infections. They focus on several successfully repurposed drugs, including former antihelminthic, anticancer, anti-inflammatory, immunomodulatory, and even psychopharmaceutical compounds. They have summarized their mechanisms and the status of on-going clinical trials for repurposed drugs like meloxicam, a widely available non-steroid anti-inflammatory drug. A different strategy, called drug redirecting, has been proposed by Jang et al. This strategy involves modifying a previously established chemical moiety to change its selectivity. Their example used an apoptotic inhibitor, YM155, that generates reactive-oxygen species upon entry into the target cells. By changing the substituents at the N3 position, they have optimized the antibacterial efficacy, possibly by selectively targeting the drug to Gram-positive bacterial cells, including MRSA.

Drug combination can improve treatment efficacy and reduce drug dosages to minimize side effects, as exemplified in the combination of β-lactams (e.g., clavulanic acid and amoxicillin or ticarcillin; Salerno and Cazzaniga, 2010). In this Research Topic, Na et al. investigated the inhibitory activity of a cellular metabolite, NADPH on the class C β-lactamases such as AmpC BER. Based on their previous study (Na et al., 2017), they presented a molecular docking model indicating that this dinucleotide could bind AmpC BER. Combination of NADPH with ceftazidime successfully restored sensitivity to ceftazidime-resistant bacteria in an experimental murine infection. Strengths of this approach include that it may not require new chemical discovery and may be combined with repurposing or repositioning efforts as well.

del Carmen Parquet et al. showed that an iron-chelating polymer (DIBI) suppressed a wound infection caused by MRSA. This makes intuitive sense, as iron is essential for microbial growth and survival. DIBI also increased the bactericidal activity of several antibiotics. DIBI also inhibits the growth of *Candida albicans* and increases its sensitivity to azole drugs *in vitro* and *in vivo* (Savage et al., 2018).

Another transition metal discussed here is zinc. Interestingly, Crane et al. report a surprising role for zinc in preventing the SOS response, which is required for the hypermutation-mediated generation of antibiotic resistance and the horizontal transfer of resistance genes. Both zinc acetate and zinc pyrithione were effective, with the latter being more active. This suggests another approach to mitigate bacterial resistance may be to compromise the pathways that generate it.

## Phage-Based Approaches

Recent advances in biotechnology have begun to allow the design, development, and production of new biologics with several advantages over chemical therapies. These include greater safety, potency, and specificity, potentially leading to fewer side effects. One “bioantibacterial” approach increasingly drawing favor is to revive the exploration of bacteriophages (phages) (Kim et al., 2019). The ability of phages to amplify at the site of infection and to cause the death and lysis of their bacterial targets makes it so that they can specifically eliminate an infectious bacterial strain or species without affecting the host microbiota (Abedon and Thomas-Abedon, 2010). In addition, as hosts develop resistance, phages may be able to evolve in concert to maintain their efficacy. Phages show substantial promise in a number of ways (Gorski et al., 2019; Hansen et al., 2019). One strategy practically at hand is to harness the endolysins produced by phages to degrade bacterial cell walls. For example, Wu et al. showed that the novel phage PD-6A3, and its purified endolysin Ply6A3, mitigated murine sepsis in approximately one-third of cases where clinical multi-drug resistant strains of *Acinetobacter baumannii* were tested. Seijsing et al. showed that extending serum retention time, such as by fusing an albumin-binding domain to the LysK endolysin from *S. aureus* phage improved efficiency.

## Other Bio-Antibacterial Approaches

In addition to the phage-based therapies mentioned above, efforts to harness naïve or engineered antimicrobial peptides (AMPs) for clinical use have been accelerating. AMPs are generally short, positively-charged peptides that contribute to the innate immune systems of a wide variety of life forms. AMPs are conventionally thought to kill microbial pathogens directly by targeting membrane functions. Unfortunately, they are also toxic toward host cells, limiting their clinical use. Research teams are currently evaluating AMPs in clinical trials as novel anti-infectives and as immunomodulators and promoters of wound healing. Here, Zharkova et al. reported that mammalian AMPs can synergize with a variety of conventional antibiotics, including fluoroquinolines, polyketides, aminogylcosides, and β-lactams. This limits the amount of AMP needed for effective treatment, and may reduce off-target effects.

Díaz-Roa et al. report identification and characterization of an AMP isolated from a necrophagous larvae, *Sarconesiopsis magellanica*. Sarconesin, as they dubbed it, showed no sign of cytotoxicity to mammalian cells and appears to increase permeability of the target membranes and bind to DNA. Moussouni et al. present a proof-of-concept study that leverages a rationally-designed synthetic version of MgtR that targets MgtC, a virulence factor required for intracellular survival of *Salmonella* and *Mycobacterium* species (Belon et al., 2015). They showed that synthetic MgtR (based on the *Salmonella* version of the protein) compromises the function of MgtC in *P. aeruginosa*, suppressing intracellular survival and biofilm formation.

Bacteriocins are another natural product produced by bacteria to limit competition in their natural environment. These peptides are often used in a form of chemical warfare against closely related species, giving them substantial potential for development as a therapy. Ghequire et al. reviewed the therapeutic potential of one class of these proteins, lectin-like bacteriocins, or Llps, which are produced by Gram-negative proteobacteria. Unlike most bacteriocins, Llps appear to catalyze killing from the cell surface instead of requiring internalization. This trait will be useful if they are leveraged as a treatment for Pseudomonads, which have active export pumps that may limit the efficacy of an imported toxin.

Corre et al. used a similar approach to identify effective natural products for limiting the growth of *Legionella pnuemophila*. By testing over 250 culturable bacterial isolates, they found a surprising variety of organisms that effectively limited *L. pneumophila* growth. Although this approach, which harnesses the natural competition in the bacterial microenvironment, is not unknown, it is very effective when looking for new treatments that are more specific than have been sought in the past.

Finally, Równicki et al., describe the design and study of a peptide nucleic acid (PNA)-based treatment that exploits the MazEF-HipBA toxin-antitoxin systems in *E. coli*. They show that antisense PNAs could effectively repress translation of the anti-toxin, causing bacterial death. They also showed that antisense PNAs could be used to stimulate the expression of the toxin-antitoxin system in the first place, improving the utility of the treatment. Promisingly, antisense PNAs did not seem to activate strong cytotoxicity in mammalian cells, although the effect of PNA-mRNA hybrids on the activation of the interferon response, the difficulties in delivery of PNAs into cells, and the ease of antimicrobial resistance emergence against a nucleotide-hybridization-based treatment remain concerns.

## Conclusions

Although the threat of antimicrobial resistance is substantial, myriad approaches to circumvent it are currently being researched. These include classical approaches, such as searching for natural products in the environment of the pathogen and more synthetic attempts, like the discovery of new compounds with previously unknown mechanisms, rationally-mutated bacterial toxins, or even small molecules designed *ab initio* based on virtual docking screens. If some of these methods can successfully translate to therapeutic options, the bacteriological apocalypse may yet be averted.

## Author Contributions

All authors listed have made a substantial, direct and intellectual contribution to the work, and approved it for publication.

### Conflict of Interest

The authors declare that the research was conducted in the absence of any commercial or financial relationships that could be construed as a potential conflict of interest.
